# Mainstream or special secondary school for the health, education, and well‐being of adolescents with Down syndrome: A systematic review

**DOI:** 10.1111/dmcn.70066

**Published:** 2025-11-04

**Authors:** Julia Shumway, Jill Ellis, Bianca Lucia De Stavola, Ruth Gilbert, Ania Zylbersztejn

**Affiliations:** ^1^ Great Ormond Street Institute of Child Health University College London London UK; ^2^ East London NHS Foundation Trust London UK; ^3^ NIHR Great Ormond Street Hospital Biomedical Research Centre Great Ormond Street Hospital for Children NHS Foundation Trust London UK

## Abstract

**Aim:**

To systematically examine evidence on the impact of attending mainstream compared with special secondary school for adolescents with Down syndrome, in terms of health, education, and well‐being outcomes.

**Method:**

We searched four bibliographic databases for studies comparing education and health (including social and self‐care) outcomes in adolescents with Down syndrome who attended mainstream secondary school to those attending special secondary school.

**Results:**

Of 4458 publications, we identified three studies from the UK and the Netherlands, which involved 246 adolescents with Down syndrome: 49 attended mainstream and 197 attended special secondary school. Of three studies examining education outcomes, two reported improved attainment among adolescents attending mainstream school, but both were at risk of bias from participant selection, missing data, and deviations to the intended intervention. One study reported social and self‐care outcomes, with no significant differences. No studies reported health outcomes. Studies provided only cursory information about teaching support.

**Interpretation:**

Parents, policy‐makers, and others who make choices about education for adolescents with Down syndrome lack evidence on whether outcomes differ, on average, between mainstream and special secondary schools. Well‐designed studies are needed to quantify the impact of secondary school type on outcomes among adolescents with Down syndrome.

## Graphical abstract

This systematic review identified three studies comparing education, health, social, and self‐care outcomes among adolescents with Down syndrome who attended either mainstream or special secondary school. Two studies reported improved education outcomes among adolescents in mainstream secondary school. Parents, educators, and policy‐makers need well‐designed quantitative studies to quantify the impact of school type on outcomes among adolescents with Down syndrome.

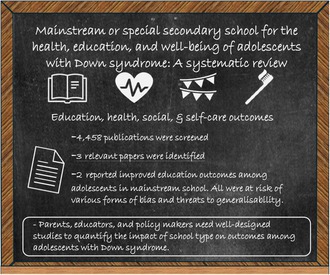

**Abbreviations**
AAI: Academic Attainment Index
ECHILD: Education and Child Health Insights from Linked Data
PICOS: Population, Intervention, Comparator, Outcome, and Study

**What this paper adds**

- Three non‐randomized studies compared outcomes among adolescents with Down syndrome by school type.
- In two studies, education outcomes improved for selected participants attending mainstream school.
- Both studies were subject to bias and limited generalizability.
- No studies reported health outcomes.

Down syndrome is the most common chromosomal cause of intellectual disability, affecting more than one in every 1000 live births.^1^, ^2^ The associated level of intellectual disability ranges from mild to profound, with frequent complication by chronic health conditions and physical disabilities including major congenital heart anomalies, low muscle tone, visual impairment, and hearing loss.^3^ The abilities of children with Down syndrome are often underestimated owing to the nature of their disability. People with Down syndrome tend to have higher receptive language than language production, so they may understand more than is reflected in their verbal responses, and their physical impairments may slow responses to requests.^4^, ^5^

That children with Down syndrome are able to improve their intellectual, physical, and social skills with appropriate education has been recognized since John Langdon Down's first clinical description of the condition in 1866.^6^ Since the 1970s, children with Down syndrome in England and Wales have had the right to state‐funded education.^7^ Still, educators, politicians, and advocates disagree about whether the most appropriate education for children with Down syndrome occurs in mainstream or special schools.^8^, ^9^, ^10^

Those who advocate for mainstream inclusion point to higher scores on tests for language, literacy, grammar, memory, and social abilities in observational studies of children with Down syndrome who attended mainstream primary schools compared with those attending special schools.^8^, ^11^ The differences between adolescents with Down syndrome according to school setting tend to decrease during secondary school, but it is difficult to ascertain whether this slowed progress is due to a failure of mainstream secondary schools to adequately support the intellectual and physical needs of children with Down syndrome^10^ or whether it is a feature inherent to Down syndrome.^9^ Filling this gap in understanding is critical because of the role secondary education has in preparing adolescents with Down syndrome for the transition to independence in adulthood.

In the past decade, England, Wales, and the USA have had major changes to education policy with the potential to improve provision for children with special needs.^12^, ^13^ Still, uncertainty remains about whether adolescents with learning impairment learn better and are healthier in mainstream or in special school settings.

A 2012 systematic review by de Graaf et al. found 26 studies reporting education outcomes for children with Down syndrome in regular and special schools. Of those 26 studies, only six reported results specifically for secondary school students (aged 11–18 years). We located four of the six citations (the two unlocated citations were a poster and a master's thesis), and only two studies reported quantitative outcomes comparing adolescents enrolled in mainstream school with those enrolled in special school. Neither study applied randomization in its study design. All other studies either focused exclusively on primary school children or reported outcomes without separate age groupings, with most participants being primary school‐aged.^14^

In this study, we aimed to address the gap in evidence around school setting, specifically for adolescents with Down syndrome. We systematically reviewed and synthesized evidence contained in studies that compared outcomes among adolescents with Down syndrome by whether they attended mainstream or special secondary school settings.

## METHOD

We aimed to assess the impact of any duration of attendance at mainstream secondary school, compared with attendance at special secondary school, on education and health (including self‐care and social health) outcomes for adolescents with Down syndrome. We asked the following two research questions. (1) How do health, education, self‐care, and social outcomes differ among adolescents with Down syndrome enrolled in mainstream secondary school compared with those enrolled in special secondary school? (2) What characteristics of mainstream and special secondary school influenced differences in outcomes?

We used the Preferred Reporting Items for Systematic Reviews and Meta‐Analyses Protocols (PRISMA‐P) guidelines to develop the study protocol (PROSPERO registration number CRD42023459964) and to guide reporting.

### Inclusion criteria

To guide our inclusion criteria for type of study design, we conducted a search (in August 2024) of the Cochrane Library, ISRCTN, clinicaltrials.gov, the World Health Organization's International Clinical Trials Registry Platform, and the EU Clinical Trials Register for randomized controlled trials, using terms for Down syndrome and school. We found no randomized controlled trials assessing placement in mainstream secondary school compared with special school for adolescents with Down syndrome. On the basis of these results, we anticipated identifying only non‐randomized comparative studies, but we made our search as broad as possible to identify any eligible randomized controlled trials.

We included studies that reported comparisons of any health, education, self‐care, or social outcome between adolescents with Down syndrome who attended mainstream secondary schools and those who attended special secondary schools. The criteria for inclusion are summarized using the Population, Intervention, Comparator, Outcome, and Study (PICOS) design criteria below.

#### Population

To ensure studies were generalizable to the wider population of adolescents with Down syndrome, we required the following: (1) more than half of study participants must have Down syndrome, or outcomes for participants with Down syndrome must be reported separately from other participants; (2) more than half of study participants must be aged 11 to 18 years, or outcomes for participants aged 11 to 18 years must be reported separately from other participants; (3) following registration of the protocol, we added a requirement that the minimum sample must have at least 10 individuals in both intervention and comparison groups. This last requirement was added to ensure study designs were robust and would allow the comparison of participants' baseline or other characteristics.

#### Intervention

Mainstream school was defined as a school setting whose population reflected the general population of students of the same age, and in which students of all abilities spent most learning and non‐learning time with their non‐disabled peers. Although we did not require a specified duration for intervention, we did require that some duration be reported.

#### Comparison

Special school was defined as any school setting in which most students had either a physical or learning disability. This included special units in mainstream schools, provided students attended these units for a majority of learning and non‐learning time.

#### Outcomes

Any measure of education, health (including physical or mental health, or health‐care contacts), self‐care, or social well‐being was included. We included only studies that reported outcomes separately for the intervention and comparison groups.

#### Study design

Randomized and non‐randomized studies of interventions were included. To ensure a robust analysis, non‐randomized studies needed to attempt to control for confounding in some way (see extraction sheets, Appendix S1).

#### Language

We did not stipulate language as an exclusion criterion.

### Search strategy

We used search terms (listed in Table S1) to describe the PICOS criteria in free text and Medical Subject Heading (MeSH) in four bibliographic databases: (1) Ovid MEDLINE (1946–2024 August week 4); (2) Scopus (inception to 2024 August week 4); (3) ERIC through EBSCOhost (1996–2024 August week 4); (4) ProQuest Dissertations and Theses Global (1861–2024 August week 4).

We first screened titles and abstracts for PICOS criteria using DistillerSR (Evidence Partners, Ottawa, ON, Canada) and EndNote (version 21; Clarivate, Philadelphia, PA, USA). JS screened all abstracts, and second reviewers (RG and AZ) checked a sample of included and twice as many excluded publications. We calculated reviewers' interrater reliability and Cohen's kappa for each pair. Reviewers resolved all discrepant inclusion decisions through consensus. After initial screening, JS additionally searched the reference lists of key reviews identified in the search.^14^, ^15^, ^16^, ^17^, ^18^, ^19^, ^20^, ^21^, ^22^, ^23^, ^24^

JS then performed a full‐text review of remaining studies to determine whether they met inclusion criteria. We used a screening tool requiring a screener to answer ‘yes’ or ‘probably yes’ for signalling questions relating to each PICOS criteria (Appendix S1). A second reviewer (BLDS) screened a sample of included and twice as many excluded papers. Reviewers discussed and resolved any conflicts through consensus with a third reviewer. JS performed an additional ‘snowball’ search of the reference and citation lists of included studies, and the steps above were repeated for identified studies.

We did not exclude any studies on the basis of language. We used Google Translate for title and abstract screening when no English‐language abstracts were available. For full‐text review, we consulted individuals at the UCL Institute of Child Health with language expertise to provide translation services.

### Data extraction

We extracted data from and assessed bias for included papers using the extraction and bias assessment tool in Appendix S1. All papers were reviewed by JS and a second reviewer (RG, AZ, BLDS, or JE). In instances where studies reported outcomes for multiple subcategories in one overall outcome domain (e.g. for the domain of self‐care, subcategories could include dressing, toileting, and self‐feeding), we reported only the overall domain score, rather than subcategories. To decrease bias in outcome measurement and reporting, after registering the protocol, we determined we would report outcome measures that were externally validated when available; when both contemporary and historical controls were included, we would report only contemporary controls.

### Risk of bias of included studies

We assessed papers' non‐randomized study designs using questions adapted from the Risk of Bias in Non‐randomised Studies – of Interventions (ROBINS‐I) tool, with reference to Thompson et al.^25^, ^26^ All papers were reviewed for risk of bias by JS and a second reviewer (RG, AZ, BLDS, or JE). We compiled a comprehensive summary of the bias affecting previous studies and outlined the caveats necessary for decision makers to apply and understand their results.

### Narrative synthesis

Owing to the substantial differences between included study designs, outcomes, and measures, meta‐analysis was not possible. Instead, we conducted a narrative synthesis addressing the four‐element general framework for narrative synthesis from the Guidance on the Conduct of Narrative Synthesis in Systematic Reviews produced by the UK's Economic and Social Research Council Methods Programme.^27^

## RESULTS

### Study selection

We used a PRISMA flowchart to summarize papers identified, screened, assessed for bias, and subjected to data extraction (Figure S1).^28^ We initially retrieved 4330 publications (Figure S1). An additional 1277 references were identified from reference lists of key reviews^14^, ^15^, ^16^, ^17^, ^18^, ^19^, ^20^, ^21^, ^22^, ^23^, ^24^ and a ‘snowball’ search of included studies' reference and citation lists. After removing duplicates, 4458 publications remained for title and abstract screening. We identified 140 publications to be screened at full‐text level. Second reviewers (RG and AZ) checked 420 titles and abstracts (9% of all publications screened by JS; 140 were included, plus twice that number excluded). Interrater reliability was high: reviewers agreed on 86% of screening decisions, with values of Cohen's kappa of 0.71 (JS and AZ; substantial agreement) and 0.48 (JS and RG; moderate agreement).^29^ Disagreements reflected caution in JS's initial screening: 39 of 48 disagreements were papers initially included by JS which second reviewers judged as exclusions; upon discussion, the nine other papers that were disagreed upon were excluded by a consensus decision.

Through full‐text review, JS identified 13 papers for assessment by a second reviewer. BLDS screened 39 papers (28% of all full‐text publications reviewed by JS; 13 were included, plus twice that number excluded), and we came to a consensus that nine of the 13 studies met the PICOS criteria. During data extraction and bias assessment, reviewers encountered several studies with design flaws. In response, we conducted an extended full‐text review, considering the study designs of each of the nine included studies. We determined, by consensus, to eliminate six studies whose design flaws made the intervention, comparison, or outcome impossible to quantify (the rationale for each exclusion is detailed in Table S2). We did not calculate interrater reliability for the full‐text screening, as the process was cautious, iterative, and all decisions were made by consensus. Three studies were included in the final synthesis.

### Characteristics of mainstream school

In all three studies summarized in Table 1, more adolescents with Down syndrome attended special school than mainstream secondary school. These studies did not provide a detailed description of mainstream schooling practices across localities, but they did give some information about classroom participation and support from teaching assistants.

**TABLE 1:** Characteristics of included studies.

Characterisitc	Buckley et al.^30^, ^32^, ^a^		Turner et al.^31^	de Graaf and van Hove^9^	
Title	A comparison of mainstream and special education for adolescents with Down syndrome: Implications for parents and teachers		Predictors of academic attainment of young people with Down's syndrome	Learning to read in regular and special schools: A follow‐up study of students with Down syndrome	
Author affiliations	The Down Syndrome Educational Trust, University of Portsmouth, National Foundation for Education Research		University of Dundee, University of Manchester, University of Stirling	Dutch Down Syndrome Association, Ghent University	
Location	Hampshire, UK		Manchester, UK	The Netherlands	
Population	Adolescents with Down syndrome attending state school		Children with Down syndrome	Children with Down syndrome	
Study design	Cross‐sectional study in 1999 and before‐and‐after comparing 1987^b^ and 1999		Prospective cohort study with 14‐year observational follow‐up (1986–2000)	Prospective cohort with 4.5‐year observational follow‐up (2006–2010)	
Sampling method	Census: 77% of all families in Hampshire in 1999, excluding five least able; 84% in 1987		Census: the original 181 included families represented 90% of all children with Down syndrome born in Manchester	Stratified random sample from within a larger random sample; 10 males and 10 females per year; with exclusions for missing IQ data	
Age distribution	11 to 20 years in 1999		Aged 6–13 years in 1986; aged 11–18 years in 1991; aged 19–26 years in 2000	Aged 9–13 years in 2006; 13.5–17.5 years in 2010	
Study size, *n*	131		71	44	
Intervention (mainstream), *n*	18		17	14	
Comparison (special), *n*	C1	23	54	C1	24
	C2	90		C2	6
Intervention description	Enrolled in mainstream school in 1999 (southeast Hampshire)		Ever attending mainstream school between 1986 and 2000	3.5–4.5 years mainstream school between 2006–2010	
Comparison description	C1	Enrolled in special school in 1999 (northwest Hampshire)	Never attended mainstream school at any point between 1986 and 2000	C1	0 years of mainstream school between 2006–2010
	C2	All adolescents enrolled in school in 1987 (all attended special schools)		C2	0.5–2.5 years of mainstream school between 2006–2010
Assessment tool	Vineland Adaptive Behaviour Scale		Academic Attainments Index (AAI)	Questionnaire assessing reading ability	
Respondents	Parents		Parents (35), tutors (31)	Parents	
Outcome description	Mean assessment scores by intervention group		Increase in AAI score from 1986 to 1991, and from 1991 to 2000	Mean difference in reading scores by intervention group	

Abbreviations: AAI, Academic Attainment Index; C1, control group 1; C2, control group 2.

^a^ In Buckley et al.,^32^ the authors provide additional details about the cohort included in their study.^30^

^b^ Because a contemporary control group was provided, we do not report outcome measures for this historical control group in Table 3.

### Duration of enrolment in mainstream schools

The duration of enrolment in mainstream secondary school varied between studies, and this variability was reflected in the classification of the intervention. While Buckley et al. classified adolescents aged 11 to 20 years enrolled in mainstream school in 1999 as having received the intervention, they reported that all but two (of 18) members of the intervention cohort had only ever attended mainstream school before 1999.^30^ De Graaf and van Hove defined the intervention group as children aged 9 to 13 years in 2006 who attended mainstream school for 3 years 6 months to 4 years 6 months during the 4 years 6 months of follow‐up. By 2010 (aged 13 years 6 months–17 years 6 months), they would have all received some mainstream secondary education, most of which was in mainstream settings.^9^

Turner et al. classified the intervention less precisely, as individuals aged 19 to 26 years who had ever attended mainstream school.^31^ Out of the 17 individuals included in the intervention group, nine students attended mainstream school for the entirety of their schooling. Two additional students finished secondary school in a mainstream setting, although they attended special school at some time during their schooling. It was impossible to know whether the remaining six individuals included in the intervention group received any mainstream schooling during secondary school.^31^

### Support from teaching assistants in mainstream schools

While few characteristics of mainstream schooling were available in the included studies, we were able to infer that level of support from teaching assistants varied by setting. Buckley et al. reported that students in mainstream secondary school were supported by dedicated teaching assistants for most of the school day.^30^ De Graaf and van Hove provided no details for study participants but reported that pupils with Down syndrome attending mainstream school in the Netherlands (without differentiating primary and secondary) received an average of 9 hours of dedicated support from teaching assistants and other special education specialists each week.^9^ Turner et al. did not report support from teaching assistants.^31^

### Classroom inclusion

Both Buckley et al. and de Graaf and van Hove reported that separating mainstream students into special classes was rare in their cohorts.^9^, ^30^ Turner et al. did not report details of classroom inclusion.^31^

### Risk of bias of included studies

We summarize the risk of bias for included studies in Table 2. All papers suffered from at least moderate risk of bias. Overall, the paper by de Graaf and van Hove suffered the lowest risk of bias, with only moderate risk for bias due to selection of participants into the study and missing data.^9^ The other two papers both suffered from moderate to high risk of bias due to selection of participants into the study, deviations from the intended intervention, missing data, and one suffered from biased measurement of outcomes.

**TABLE 2:** Bias assessment, according to ROBINS‐I domains, classified as low risk, moderate risk, and high risk.

Risk of bias and details	Buckley et al.^30^, ^32^, ^a^	Turner et al.^31^	de Graaf and van Hove^9^
Bias due to confounding by a time‐fixed exposure	High risk	Low risk	Low risk
	There were differences in characteristics of the two groups that were not accounted for in statistical comparisons: the special school group was, on average, 2 years older than the mainstream group; a higher proportion of females than males attended mainstream school, and the sex distribution was uneven by age; school type was assigned largely on the basis of location, which may be related to social and income deprivation.	While the authors showed the highest predictor of AAI score at time 3 (year 2000; ages 19–26) was AAI at times 1 (year 1986; ages 6–13) and 2 (year 1991; ages 11–18), they also measured and accounted for child and family characteristics which may affect a child's access to mainstream school as well as their AAI score at time 3.	An extensive list of potential confounders was measured and included in the analysis of covariance, which compared outcomes.
Bias in selection of participants into the study	High risk	Moderate risk	Moderate risk
	While authors report that non‐participating students were similar to participants, no characteristics of non‐participants were reported. The authors excluded the five students attending special school who had the lowest ability, reasoning that they would never have attended mainstream school. No characteristics of these students were reported. The younger‐age makeup of the intervention group suggests older students may have transferred to special school or left school.	Children were excluded for low or missing IQ tests at time 1 (1986). Authors report and account for many causes of attrition, but participants with higher abilities may have stayed in the study longer.	The response rate for 2010 was 95%; 14% of 2010 respondents were excluded owing to lack of IQ report.
Bias in classification of interventions	Low risk	High risk	Low risk
	Authors' close work with schools allowed accurate classification.	Of the 17 who ever attended mainstream school at any time during primary or secondary school, there is no report of whether six attended any mainstream secondary school.	It was possible to compare those who had spent most of their time in mainstream school (including those who had only transferred to secondary school in the past year) against those who had either some or no mainstream school experience.
Bias due to deviations from intended intervention	Moderate risk	High risk	Low risk
	Authors report four students switching school type (two from special to mainstream and two from mainstream to special) at the transition from primary to secondary school over the course of 9 years; it is unclear whether these students were study participants, or whether any additional switching occurred during secondary school.	No report of classrooms or teaching aids in mainstream schools or school experiences. Inconsistent report of the duration of or age at which the intervention was received.	Authors reported and accounted for deviations.
Bias due to missing data	Moderate risk	Moderate risk	Moderate risk
	Outcome data were missing for two participants (one from each group) without explanation of how missing data were addressed.	High levels of attrition. Participants with higher abilities may have stayed in the study longer, leading to an exaggeration of the intervention's effect.	Of 2010 respondents, 14% were excluded owing to failure to provide an IQ report. Owing to this missing data, the least able children may be excluded from the analysis.
Bias in measuring outcomes	Low risk	Moderate risk	Low risk
	All assessments were conducted by parents, which is considered valid for the Vineland Adaptive Behaviour Scales.	The Academic Attainment Index was designed for teacher assessment, but parents conducted 35 of 71 assessments at time 3. Possibly because they filled in more of the checklist items, scores were higher when reported by parents compared with tutors. Still, previous parent scores had been validated against teacher scores and showed no evidence of bias.	All assessments were conducted by parents. While parents may have been biased in their assessments, authors validated against teachers' assessments. Authors addressed a possible ceiling effect with too low of a scale for the outcome measure.
Bias in selection of reported result	Low risk	Low risk	Low risk
	Authors reported all planned outcome measures.	Authors reported all planned outcome measures.	Authors reported all planned outcome measures.

*Note*: Level of bias is colour‐coded, from dark red (high risk) to light blue (low risk).

Abbreviations: AAI, Academic Attainment Index; ROBINS‐I, Risk of Bias in Non‐randomised Studies – of Interventions.

^a^ In Buckley et al.,^32^ the authors provide additional details about the cohort included in their study.^30^

A moderate risk of bias was related to unaddressed confounding factors associated with both school type and outcomes in the paper by Buckley et al. because the mainstream school group was systematically different from the special school group: the mainstream group had a 2‐year lower mean age and a 17% higher proportion of females. Additionally, assignment to school type was partly based on the geography of the child's residence, which is related to social, income, and class‐related factors which were not accounted for and may have influenced results.^30^ Turner et al. controlled for a variety of family‐ and child‐related characteristics, as well as previous Academic Attainment Index (AAI) scores and an initial assessment of ‘mental age’ in their model comparing educational outcomes. Authors acknowledged that pre‐existing ability may have been a confounder, as children with the highest initial AAI scores remained in mainstream school longest.^31^ De Graaf and van Hove similarly measured a range of potential social and demographic confounders at baseline and adjusted their models accordingly.^9^

All three papers were at risk of selection bias which would affect the generalizability of their findings. The paper by Buckley et al. was at highest risk: the authors reported contacting 95% to 100% of all families of children with Down syndrome in their study location (the county of Hampshire, UK), and that 77% of those contacted participated. While the authors reported their belief that the adolescents and families who declined to participate did not vary ‘in any significant ways’ from study participants, no characteristics were formally measured or reported.^32^ Additionally, the five ‘least able’ adolescents attending special secondary schools were excluded as they ‘would not have been placed in mainstream classes in any part of the county’. While the authors reported some characteristics (of note: four of the five excluded were male), they reported no measures of these adolescents' abilities.^30^ Both Turner et al. and de Graaf and van Hove excluded potential participants for missing IQ scores,^9^ and Turner et al. also excluded those with the lowest IQ scores.^31^

The paper by Turner et al. was at high risk for classification bias because participants were grouped on the basis of whether they ever attended mainstream primary or secondary school at any point between 1986 and 2000. The intervention group comprised 17 participants who had ever attended mainstream school in the 14‐year study period. Nine of these participants had attended mainstream school exclusively during primary and secondary school. Two participants' final secondary school enrolment was in mainstream school, but they had attended special school at some point, making it unclear whether all or even most of their secondary school experience was mainstream. For the other six participants who had ‘some mainstream schooling’ in the 14‐year study period, it was impossible to ascertain whether any of this mainstream experience took place during secondary school or whether it occurred entirely during primary school.^31^

All three papers were subject to a moderate risk of bias due to missing data. Turner et al. reported high levels of attrition over the course of follow‐up;^31^ de Graaf and van Hove excluded 14% of respondents from their study owing to missing IQ data;^9^ in Buckley et al., outcome measures were missing for two students (one from each arm of the study), but these missing data were not addressed.^30^

The paper by Turner et al. suffered from bias in measuring outcomes: some participants' outcomes were measured by parents and some by tutors. While the authors had validated previous parental measures of AAI scores against teachers' assessments, they report that, at time 3, parents' assessments were both more complete and higher on average than tutors' assessments. Authors also reported that scores were higher when measured by parents.^31^ In contrast, de Graaf and van Hove did not suffer the same source of bias because all assessments were conducted by parents, and the authors validated parents' assessments against teachers' assessments and found measures to be comparable.^9^ In Buckley et al., all questionnaires were completed by parents.^30^

### Funding sources

Although none of the papers provided explicit funding statements, each did list the authors' affiliations, which we show in Table 1.

### Effect of mainstream vs special secondary school on outcomes

We summarize the effect of mainstream and special secondary school on outcomes reported in the three studies in Table 3. All three studies examined education outcomes, one study reported social and self‐care outcomes, and no studies reported health outcomes.

**TABLE 3:** Summary of estimated effect of mainstream compared with special secondary school on education, social, and self‐care outcomes extracted from three included studies.

Study	Outcomes						
	Education			Social		Self‐care	
Buckley et al.^30^	Vineland Total Communication Skills, mean score (SD)			Vineland Total Socialisation Skills, mean score (SD)		Vineland Total Daily Living Skills, mean score (SD)	
	Mainstream	Special		Mainstream	Special	Mainstream	Special
	**90.17 (40.63)**	**51.59 (18.56)**		74.94 (29.06)	87.50 (55.46)	76.82 (24.10)	78.13 (40.43)
	Differences in outcomes between intervention groups were tested using a two‐way analysis of variance						
Turner et al.^31^	Increase in Academic Attainments Index score, with *p*‐value for *t*‐test			—		—	
	Value at t2, controlled for value at t1	Value at t3, controlled for value at t2		—		—	
	**1.55 (*p* = 0.13) beta weight 0.16**	**3.65 (*p* < 0.001) beta weight 0.19**		—		—	
de Graaf and van Hove^9^	Mean difference from 2006 to 2010 in reading score			—		—	
	I	C1	C2	—		—	
	4.0 (3.0)	4.0 (4.3)	2.7 (2.9)				

*Note*: Statistically significant results (*p* < 0.05) are in bold type.

Abbreviations: C1, first control group, 0 years in mainstream school; C2, second control group, 0.5–2.5 years in mainstream school; I, intervention group; SD, standard deviation; t1, t2, t3, prospective study waves.

Of three studies examining education outcomes, Buckley et al. and Turner et al. reported statistically significant results for assessments of academic ability: in Buckley et al., adolescents enrolled in mainstream school scored higher, on average, than those attending special school;^30^ and the analysis in Turner et al. estimated that adolescents who had ever attended mainstream school would have a 3.65 higher AAI score at time 3 than those who had not in a 9‐year period, even after controlling for their time 2 score.^31^ The authors supported these findings with a path analysis which accounted for the correlation coefficients of the direct and indirect effects of covariates and potential confounders. In contrast, de Graaf and van Hove reported no significant differences between mainstream and special school groups, measured as the change in scores over a 4.5‐year period.^9^

Buckley et al. were the only authors to examine social and self‐care outcomes, and they measured and reported multiple outcomes for domains and subdomains from the Vineland Adaptive Behaviour Scale (made up of three domains and nine subdomains) as well as the Buckley and Sacks Questionnaire (made up of four domains and 14 subdomains), which was created by the authors. We report Vineland Adaptive Behaviour Scale measures in Table 3 because they were externally validated, and we report the overall domain scores, rather than contributing subdomains, some of which did show significant differences between mainstream and special school groups.^30^ We do not report the comparison between the 1999 mainstream and secondary students against the historical 1987 special school students because of the potential for unaccounted differences in the collection of data, composition of the 1987 cohort, demographic shifts, and changes in the educational practices over time.^32^

### Generalizability

The comparison and generalizability of the three papers are subject to differences between the Dutch and English secondary school systems. Both the papers by Turner et al. and de Graaf and van Hove controlled for several child‐ and family‐level covariates and addressed causes for missing data due to attrition or incomplete parent reports which might have affected the generalizability of their findings. In each case, missing data were likely to be related to lower academic performance. Therefore, their findings may not be generalizable to adolescents with Down syndrome who have the lowest levels of pre‐existing ability.^9,30,31^

The paper by Buckley et al. suffered from missing data and from lack of adjustment for potential confounders. Additionally, the generalizability of the findings is limited because the intervention may have differed substantially from the widespread implementation of mainstream schooling for adolescents with Down syndrome: the authors were associated with the Down Syndrome Educational Trust, UK, which they state ‘pushed for inclusion … in our locality …’.^32^ The Trust also ‘support[ed] the schools … [visiting no more] than once a term, on average, unless asked to help with a problem’.^30^ This level of dedicated support for mainstream schools serving adolescents with Down syndrome is unlikely to be replicated on a general level.

## DISCUSSION

In this systematic review, we analysed the impact of mainstream compared with special secondary school on outcomes for adolescents with Down syndrome. We found only three studies that reported results for secondary school placement, two of which (Buckley et al. and Turner et al.) had been included in the 2012 systematic review by de Graaf et al.^14^, ^30^, ^31^ We assessed these studies for bias and found that the reported effect of mainstream secondary schooling on improved academic outcomes may be partly explained by unmeasured confounding due to ability or attrition of less able pupils. The paper by de Graaf and van Hove, with its robust study design and lower risk of bias, reported no difference in attainment.^9^ It is worth noting that no studies reported a deleterious effect of mainstream schooling on academic or other outcomes.^9^, ^30^, ^31^ Only Buckley et al. reported social and self‐care outcomes among adolescents aged 11 to 20 years, but the overall domain scores showed no significant differences by secondary school type.^30^

There were several limitations of the evidence. The included studies all suffered from some moderate levels of bias. All three studies were limited by at least a moderate risk of bias due to selection of participants into the study and missing data. The two papers which reported improved education outcomes (Buckley et al. and Turner et al.) also suffered from a moderate to high risk of bias due to deviations from the intervention and measuring of outcomes.^30^, ^31^

No identified studies quantitatively addressed our second research question: what characteristics of mainstream and special secondary school influenced differences in outcomes? Only limited qualitative information was provided to explain which school characteristics influenced differences in outcomes. It is possible that higher attainment among adolescents in mainstream schools related to individual child support, particularly from teaching assistants, whose support was mentioned in two studies (Buckley et al. and de Graaf and van Hove).^9^, ^30^ However, there was insufficient reporting of support in either mainstream or special school settings, particularly on the type and frequency of support from teaching assistants.

We also found little detailed information about how or where time in mainstream school had been spent. We had defined the intervention in this review broadly, to include either special secondary schools or special units in mainstream secondary schools. But we found no studies that compared outcomes among students attending different types of classrooms or units in mainstream schools.

The study by Buckley et al. is the only one to provide any reason why a certain type of school placement was chosen (geography).^30^ While the baseline characteristics provided in the studies by de Graaf and van Hove and Turner et al. allowed researchers to adjust statistically for certain factors, future research would benefit from an ability to quantify how children's needs may have influenced school placement.^9^, ^30^

Finally, although we did not exclude studies on the basis of location, all included studies were conducted in high‐income nations, and their findings may not be applicable to other settings.

Researchers can address many of these limitations by designing future studies to examine how the impact of school setting can improve their papers' external validity by collecting and reporting more detailed information about the types and levels of support provided in each type of school. All studies should report children's characteristics as well as baseline measures for outcomes. Future studies should also report baseline health characteristics, a range of self‐care and social outcomes, and consider measures of physical health outcomes. Ideally, randomization would create comparison groups balanced for children's characteristics and learning needs, but randomized controlled trials of school setting may not be feasible, owing to potential conflicts with children's needs and family preferences. Some limitations can be overcome by using population‐level administrative data in future research. Such data are becoming increasingly available; for example, the Education and Child Health Insights from Linked Data (ECHILD) project provides school records from the National Pupil Database linked to Hospital Episode Statistics. Hospital Episode Statistics can be used to develop representative cohorts of children with Down syndrome, following them longitudinally from birth.^33^ Through ECHILD, these cohorts can be joined to data on school placement available in the National Pupil Database, with multiple outcomes and covariates measured at baseline and tracked over time.^34^ Applying robust causal inference methods to these data presents researchers with the novel opportunity to quantitatively measure the influence of secondary school type on education, health, and other outcomes in adolescents with Down syndrome.

Multiple methods of research are necessary to improve our understanding of health and education outcomes in adolescents with Down syndrome. Bias could be minimized through randomized or cluster‐randomized study designs linked to existing administrative data. Our understanding of pupils' experiences will also benefit from detailed data on classroom activities and characteristics that are not currently available in ECHILD. Further information on class size and the role of teaching assistants in the classroom would provide context for understanding the actual pedagogy provided to students.^35^, ^36^ Additionally, we will be better able to interpret the results of quantitative research when we put results into context with qualitative research and public engagement and involvement with families and adolescents with Down syndrome.^37^

In systematically screening papers for inclusion in this review, we encountered many papers on the topic of parents' experiences,^38^, ^39^ teachers' attitudes,^40^, ^41^ and the experiences and attitudes of the peers^42^, ^43^ of children with Down syndrome. This orientation of research towards parents, teachers, and peers implies that the motivation for the school placement of adolescents with Down syndrome is not based solely on the best academic interests of the individual student but also on the needs and views of parents, teachers, and other students. Which raises the question, what is the purpose of schooling for adolescents with Down syndrome? Is it to give them marketable skills? Is it to embed them in a community? Is it to enrich them personally with ideas from history, great literature, science, and mathematics?

As part of this research, we met with parents of adolescents with Down syndrome from regions across England. Parents have shared three key motivations for where their children are enrolled in school: (1) allowing their child to be part of the local community; (2) meeting their child's health and intellectual needs; and (3) surrounding their child with a supportive culture of teachers and other students.^44^

Parental interviews and available research tell the same story: for parents of adolescents with Down syndrome, culture and community are at least as important as the academic environment in which an adolescent will be educated.^21^, ^44^ It is for this reason that future research on the educational placement of adolescents with Down syndrome must include quantitative measures of not only education outcomes, but also classroom characteristics, mental and physical health, self‐care skills, and social well‐being of children and their families.

## CONCLUSION

There remains a gap in research comparing outcomes between adolescents with Down syndrome who attend mainstream secondary school with those who attend special secondary school. To date, most research comparing outcomes among children with Down syndrome who attend mainstream versus special school has focused on primary school children, and all identified research on adolescents attending secondary school has been conducted in high‐income countries. Those studies that have compared adolescents with Down syndrome by secondary school placement have suffered from bias, particularly selective recruitment, bias due to missing data, and attrition bias.

Among the studies we did identify, the outcomes do not necessarily align with the outcomes valued by families.^21^, ^44^ Beyond academic goals, parents value community, health, and happiness in their children. Parents want their children to be happy and engaged in school; they shared that skin conditions, stomach problems, sleep apnoea, and behavioural problems manifest at times of poor mental health.^44^ The relationship between school enrolment and specific health and behavioural patterns should be studied, particularly as children with Down syndrome are likely to have complex health‐care needs. Parents also want their children to feel a part of a community, with name recognition in the community and time spent socializing as important measures for future studies.

The education needs of adolescents with Down syndrome merit further study. Parents and educators need an evidence‐based understanding of which school will best suit different pupils with Down syndrome. Current advice is based on inadequate research. Well‐designed randomized and non‐randomized studies are needed to quantify the impact secondary school type has on outcomes among adolescents with Down syndrome. Such studies can be designed using population‐level, linked administrative data. These future studies will have a greater impact as they will include robust quantitative measures of classroom characteristics, education, health, self‐care, and social well‐being. Future advice should be based on robust quantitative research interpreted critically in the context of classroom experiences.

## FUNDING STATEMENT

JS was funded by the Down's Syndrome Association. Additional support was provided by the National Institute for Health and Care Research (NIHR) through the Programme Development Grant (NIHR206946), the GOSH Biomedical Research Centre (AZ), and a senior investigator award (RG). This research benefits from and contributes to the NIHR Children and Families Policy Research Unit but was not commissioned by the NIHR Policy Research Programme. The views expressed are those of the author(s) and not necessarily those of the NIHR or the Department of Health and Social Care.

## Supporting information


**Appendix S1:** Data Extraction Form for full‐text Screening and Extraction.

**Figure S1:** PRISMA flow diagram of study selection.

**Table S1:** Overview of search terms and number of records returned at each step.

**Table S2:** Studies excluded during extended full‐text review.

## Data Availability

No research data are shared.
