# Supplementary material for: Mainstream or special secondary school for the health, education, and well‐being of adolescents with Down syndrome: A systematic review
Source: Dev Med Child Neurol. 2025 Nov 4;68(6):755–66. doi: 10.1111/dmcn.70066 (PMC13160392; doi:10.1111/dmcn.70066)
Supplement: Supplementary file 1 — Appendix S1: Data Extraction Form for full‐text Screening and Extraction. [file DMCN-68-755-s002.docx]

**Appendix S1. Data Extraction Form for full-text Screening and Extraction**

Adapted from Cochrane Airways Data collection form-RCTs and NRS, with reference to Almossawi, et al., 2021.

**Notes on using study eligibility and data extraction form:**

- Be consistent in the order and style you use to describe the information for each report.
- Record any missing information as unclear or not described, to make it clear that the information was not found in the study report(s), not that you forgot to extract it.
- Include any instructions and decision rules on the data collection form, or in an accompanying document. It is important to practice using the form and give training to any other authors using the form.

| Review title or ID |  |
| --- | --- |
| Study ID *(surname of first author and year first full report of study was published e.g. Smith 2001)* |  |
| Report ID |  |
| Report ID of other reports of this study including errata or retractions |  |
| Notes | |

**General Information**

| Date form completed *(dd/mm/yyyy)* | |  |
| --- | --- | --- |
| Name/ID of person | |  |
| Screening | Extracting |  |
| Checking | Checking |  |
| Study author contact details | |  |
| Publication type *(e.g. full report, abstract, letter)* | |  |
| Notes: | |  |
|  | | |

**Study eligibility and classification**

A study must meet the requirements for each of the study criteria listed below. If the answer to any eligibility criteria question is No/Probably No or Unclear, **DO NOT PROCEED**. Report the study citation and exclusion rationale in a table for studies excluded in Stage 3.

| Study Characteristics | Eligibility criteria | Eligibility criteria met?  (Yes, Probably Yes, No, Probably No) | | | | Location in text or source *(pg & ¶/fig/table/other)* |
| --- | --- | --- | --- | --- | --- | --- |
|  |  | Yes/ PY | No/PN | | Unclear |  |
| Participants | Children with Down Syndrome* |  |  | |  | *Either a majority of participants have Down Syndrome, or a separate analysis was conducted for those with Down Syndrome |
|  | Proportion of children with DS: |  | | | |  |
|  | Aged* 12 to 18 |  |  |  | | Either a majority are aged 12 to 18, or a separate analysis was conducted for that age group. |
|  | Age makeup of participants: |  | | | |  |
| Intervention | Mainstream school |  |  |  | |  |
|  | Details regarding student population and time allotment. |  | | | |  |
| Comparison | Special school |  |  |  | |  |
|  | Details regarding student population and time allotment. |  | | | |  |
| Types of outcome measures (tick all that apply) | Educational |  |  |  | |  |
|  | List outcomes: |  | | | |  |
|  | Self-care |  |  |  | |  |
|  | List outcomes: |  | | | |  |
|  | Mental health |  |  |  | |  |
|  | List outcomes |  | | | |  |
|  | Physical heath |  |  |  | |  |
|  | List outcomes: |  | | | |  |
| Study Design | 1. Were there both an intervention and a comparator? |  |  |  | |  |
|  | How were they allocated |  | | | |  |
|  | 2. Were outcome data available after the intervention? |  |  |  | |  |
|  | At what point(s) in the study? For the same individuals? |  | | | |  |
|  | 3. Did the researchers aim to control for confounding? |  |  |  | |  |
|  | At the level of design or analysis? |  | | | |  |
|  | 4. Were groups of individuals or clusters formed on the basis of some pre-outcome variable? |  |  |  | |  |
|  | By which process? |  | | | |  |
|  | 5. Was the intervention effect estimated? Or is sufficient information provided that an effect measure can be calculated? |  |  |  | |  |
|  |  | If the answer is No/ PN, but the study meets all other criteria,contact authors to request data. Then re-assess. | | | |  |
|  | By change over time? By difference between groups? |  | | | |  |
| INCLUDE | | | | EXCLUDE | | |
| Reason for exclusion |  | | | | | |
|  | | | | | | |

**DO NOT PROCEED IF STUDY EXCLUDED FROM REVIEW**

**Additional questions for study classification**

| Study Characteristics | Eligibility criteria | Eligibility criteria met?  (Yes/Probably Yes, No/Probably No) | | | Location in text or source *(pg & ¶/fig/table/other)* |
| --- | --- | --- | --- | --- | --- |
|  |  | Yes/ PY | No/PN | Unclear |  |
| Questions for inclusion and study categorisation | 6. Were the following features of the study carried out after the study was designed: |  | | |  |
|  | 1. characterization of individuals/clusters before intervention? |  |  |  |  |
|  | 1. actions/choices leading to an individual/cluster becoming a member of a group? (d) |  |  |  |  |
|  | 1. assessment of outcomes? |  |  |  |  |
|  | Details: |  | | |  |
|  | 7. Were the following variables measured before intervention: |  | | |  |
|  | 1. potential confounders? |  |  |  |  |
|  | 1. outcome variable(s)? |  |  |  |  |
|  | Details: |  | | |  |
| Study design |  | | | | |
| **Notes:** | | | | | |

**Characteristics of included studies**

**Methods**

|  | **Descriptions as stated in report/paper** | | **Location in text or source** *(pg & ¶/fig/table/other)* |
| --- | --- | --- | --- |
| **Aim of study** |  | |  |
| **Study design** |  | |  |
| **Geographic Location** |  | |  |
| **Unit of allocation** *(by individuals, schools, class)* |  | |  |
| **Start date** |  | |  |
| **End date** |  | |  |
| **Duration of participation** *(from recruitment to last follow-up)* |  | |  |
| **Ethical approval needed/ obtained for study** | Yes No Unclear |  |  |
| **Notes:** | | | |

**Emulated Target Trial**

|  | **Descriptions as stated in report/paper** | **Location in text or source** |
| --- | --- | --- |
| **Recruitment** |  |  |
| **Eligibility** |  |  |
| **Exclusions** |  |  |
| **Treatment strategies** |  |  |
| **Outcomes** |  |  |
| **Follow-up** |  |  |
| **Causal contrast** |  |  |
| **Estimands** |  |  |

**Participants**

|  | Description (*Include comparative information for each intervention or comparison group if available* | | Location in text or source *(pg & ¶/fig/table/other)* |
| --- | --- | --- | --- |
| Population description *(from which study participants are drawn)* |  | |  |
| Setting *(including location and social context)* |  | |  |
| Inclusion criteria |  | |  |
| Exclusion criteria |  | |  |
| Method of recruitment of participants *(e.g. phone, mail, school records)* |  | |  |
| Informed consent obtained | Yes No Unclear |  |  |
| Sample size |  | |  |
| Clusters *(e.g., county, region, LE* |  | |  |
| Baseline imbalances |  | |  |
| Withdrawals and exclusions |  | |  |
| Age range |  | |  |
| Sex |  | |  |
| Race/Ethnicity |  | |  |
| Learning Disability |  | |  |
| Co-morbidities |  | |  |
| Other relevant sociodemographics |  | |  |
| Subgroups measure |  | |  |
| Subgroups reported |  | |  |
| Notes: | | | |

**Intervention groups**

*Copy and paste table for each intervention and comparison group*

**Intervention Group 1**

|  | Description as stated in report/paper | Location in text or source *(pg & ¶/fig/table/other)* |
| --- | --- | --- |
| Group name |  |  |
| No. of students in group |  |  |
| Theoretical basis *(include key references)* |  |  |
| Description *(include sufficient detail for replication, e.g., student composition, curriculum, teaching staff, SEND)* |  |  |
| Duration of attendance |  |  |
| Timing *(e.g. student age or year in school)* |  |  |
| Allocation *(e.g. how student was placed in school)* |  |  |
| Co-interventions |  |  |
| Details regarding intervention switching or cessation. *(e.g. school transfers, exclusions, absences)* |  |  |
| Notes: | | |

**Outcomes**

*Copy and paste table for each outcome.*

**Outcome 1**

|  | Description as stated in report/paper | | Location in text or source *(pg & ¶/fig/table/other)* |
| --- | --- | --- | --- |
| Outcome name |  | |  |
| Time points measured *(specify whether from start or end of intervention)* |  | |  |
| Time points reported |  | |  |
| Outcome definition *(with diagnostic criteria if relevant)* |  | |  |
| Person measuring/ reporting |  | |  |
| Unit of measurement *(if relevant)* |  | |  |
| Scales: upper and lower limits *(indicate whether high or low score is good)* |  | |  |
| Is outcome/tool validated? | Yes No Unclear |  |  |
| Imputation of missing data *(e.g. assumptions made for ITT analysis)* |  | |  |
| Assumed risk estimate *(e.g. baseline or population risk noted in Background)* |  | |  |
| Power *(e.g. power & sample size calculation, level of power achieved)* |  | |  |
| Notes: | | | |

**Other**

| **Study funding sources** *(including role of funders)* |  |  |
| --- | --- | --- |
| **Possible conflicts of interest** *(for study authors)* |  |  |
| **Notes:** | | |

**Risk of Bias assessment**

*(See the ROBINS-I Tool and Table 6, Risk of Bias Assessment Rubric to assess bias according to the following seven domains)*

| Domain | Risk of bias | | | Support for judgement  *(include direct quotes where available with explanatory comments)* | Location in text or source *(pg & ¶/fig/table/other)* |
| --- | --- | --- | --- | --- | --- |
|  | Low | High | Unclear |  |  |
| Pre-intervention | | | | | |
| 1: Bias due to confounding by a time-fixed exposure |  |  |  |  |  |
|  | Reviewers will assess how well confounding variables that could impact health outcomes were addressed in the analysis. We will consider each of the questions under Domain 1 in the ROBINS tool in order to assess whether the confounding domains listed in the ETT were addressed at the time of intervention. | | | | |
|  | Example: Deprivation may be a predictor of which school a child attends and of a child's health and academic outcomes. Reviewers will assess how well a study design addresses participant deprivation. | | | | |
| 2: Bias in selection of participants into the study |  |  |  |  |  |
|  | Reviewers will address selection bias using the alternative ROBINS-I 2.1 question proposed by Thomson, et al. (2018): "Was the sample in the final analysis different to original study sample with respect to outcome or exposure to the intervention (aside from attrition)?" In effect, they will ask whether there are features of study design or implementation that affect how well the intervention and comparison groups represent the groups the researchers intended to study. This may be affected by whether participants are classified at the time of intervention assignment or post-hoc, at the time of analysis.^18^ | | | | |
|  | Example: Studies which assign children to intervention or comparison groups post-hoc may fail to capture those students who began in mainstream school and then switched to special school. Reviewers will assess how well a study addresses changes to children's initial secondary school placement. | | | | |
| At intervention | | | | | |
| 3: Bias in classification of interventions |  |  |  |  |  |
|  | In asking whether the intervention was clearly defined, reviewers will assess whether researchers addressed school environment, peers, and teaching tools, and how similar the mainstream educational experience received by children with Down Syndrome was to the experience of their non-disabled peers. | | | | |
|  | Example: Children who attend school in the same building as their non-disabled peers may, in practice, be segregated for large parts of the day to special classrooms. This may make the intervention more similar to the comparison. Reviewers will assess how well a study defines details of the intervention and how it differs from the comparison. | | | | |
| Post-intervention | | | | | |
| 4: Bias due to deviations from intended intervention |  |  |  |  |  |
|  | There may be great variability in co-interventions and whether they are, in fact, inherent to the intervention. Reviewers will assess how co-interventions were classified, in addition to features of the intervention and comparison. Additionally, we will assess how well any legitimate co-interventions were balanced across groups, and whether any differences in implementation were addressed. | | | | |
|  | Example: A dedicated teaching aid may be an integral the implementation of mainstream schooling. But if a phonics curriculum were being implemented as a co-intervention in both intervention and control groups, it is possible a dedicated teaching aid in mainstream school may lead to the co-intervention being implemented differently in mainstream school as compared to special school. | | | | |
| 5: Bias due to missing data |  |  |  |  |  |
|  | Missing data may contribute to selection bias (e.g., attrition and immortal time bias). This would particularly be the case when participants' intervention status is classified post hoc. Reviewers will use the ROBINS-I signalling questions to assess how well the study addressed missing information about intervention status, outcome status, and any other missing data. | | | | |
|  | Example: Some children may leave state school, e.g., due to deteriorating health or in order to attend private school. If children leave before follow-up begins, researchers will be unable to determine whether the phenomena occurred at a higher rate in the intervention or comparison groups. | | | | |
| 6: Bias in measuring outcomes |  |  |  |  |  |
|  | Due to the nature of the intervention, blinding of participants and assessors will be unlikely, and some difference in how outcomes are measured between mainstream and special school may be unavoidable. Reviewers will assess whether and how studies address any differences in how outcomes are measured across intervention and comparison groups. | | | | |
|  | Example: If a standardised tests is used to assess education levels in children with Down Syndrome, it is possible children in mainstream school would be able to complete the exam in the company of peers. Children in special school would likely need such a test administered especially for purposes of the study. This difference in method for measuring the outcome may impact the children's scores and needs to be addressed by researchers. | | | | |
| 7: Bias in selection of the reported result |  |  |  |  |  |
|  | In a study with multiple outcomes of interest and effect measures, it is possible that selectively reporting the most interesting or impressive measures or analytic methods could lead to a misrepresentation of results. Thus, reviewers will assess whether a study included an advance plan for analysis and the effect measures to be reported and whether researchers adhered to their plan. | | | | |
|  | Example: A study assessing self-care skills (e.g., dressing and toileting) among children with Down Syndrome in different school settings might conduct an analysis which controls for level physical disability and one that does not. A failure to pre-specify which analysis will be reported, researchers may be tempted to selectively report the effect measure which shows the intervention at greatest advantage. | | | | |
| Other bias |  |  |  |  |  |
| Notes: | | | | | |

Data and analysis

*Copy and paste the appropriate table for each outcome, including additional tables for each time point and subgroup as required.*

|  | Description as stated in report/paper | | | | | Location in text or source *(pg & ¶/fig/table/other)* |
| --- | --- | --- | --- | --- | --- | --- |
| Comparison |  | | | | |  |
| Outcome |  | | | | |  |
| Subgroup |  | | | | |  |
| Time point *(specify from start or end of intervention)* |  | | | | |  |
| Post-intervention or change from baseline? |  | | | | |  |
| No. participants | Intervention | | | Control | |  |
|  |  | | |  | |  |
| Results | Intervention result | SE *(or other variance, specify)* | | Control result | SE *(or other variance, specify)* |  |
|  |  |  | |  |  |  |
|  | Overall results | | | SE *(or other variance, specify)* | |  |
|  |  | | |  | |  |
| Any other results reported |  | | | | |  |
| No. missing participants |  | | |  | |  |
| Reasons missing |  | | |  | |  |
| No. participants moved from other group |  | | |  | |  |
| Reasons moved |  | | |  | |  |
| Unit of analysis *(individuals, cluster/ groups or body parts)* |  | | | | |  |
| Statistical methods used and appropriateness of these |  | | | | |  |
| Reanalysis required? *(specify)* | Yes No Unclear | |  | | |  |
| Reanalysis possible? | Yes No Unclear | |  | | |  |
| Reanalysed results |  | | | | |  |
| Notes: | | | | | | |

**Other information**

|  | **Description as stated in report/paper** | **Location in text or source** *(pg & ¶/fig/table/other)* |
| --- | --- | --- |
| **Key conclusions of study authors** |  |  |
| **References to other relevant studies** |  |  |
| **Correspondence required for further study information** *(from whom, what and when)* |  | |
| **Notes:** | | |
