# Supplementary material for: Mainstream or special secondary school for the health, education, and well‐being of adolescents with Down syndrome: A systematic review
Source: Dev Med Child Neurol. 2025 Nov 4;68(6):755–66. doi: 10.1111/dmcn.70066 (PMC13160392; doi:10.1111/dmcn.70066)
Supplement: Supplementary file 2 — Figure S1: PRISMA flow diagram of study selection. [file DMCN-68-755-s001.docx]

**Figure S1**. **PRISMA flow diagram of study selection**

4,330 records retrieved in database search

137 excluded based on full-text screening (primary reason)

- 54 incorrect study design
- 36 incorrect population
- 36 incorrect intervention
- 7 full-text could not be found
- 4 incorrect outcome

3 included studies

1,277 additional records:

- 955 records referenced in relevant reviews
- 248 records citing and referenced by included studies

5,557 total records retrieved

4,458 records after deduplication

4,458 records screened

4,318 records excluded based on title and abstract screening

140 full-text records screened
