# Supplementary material for: Mainstream or special secondary school for the health, education, and well‐being of adolescents with Down syndrome: A systematic review
Source: Dev Med Child Neurol. 2025 Nov 4;68(6):755–66. doi: 10.1111/dmcn.70066 (PMC13160392; doi:10.1111/dmcn.70066)
Supplement: Supplementary file 3 — Table S1: Overview of search terms and number of records returned at each step. [file DMCN-68-755-s003.docx]

**TABLE S1** Overview of search terms and number of records returned at each step

| **Database** | **Search terms** | | **Number of records** |
| --- | --- | --- | --- |
| Ovid MEDLINE  1946 to 2024 August week 4 | 1 | Down Syndrome/ | 26 989 |
|  | 2 | (‘Down syndrome’ or ‘Down’s Syndrome’ or ‘trisomy-21’ or mongol*).mp. | 50 931 |
|  | 3 | 1 or 2 | 50 931 |
|  | 4 | education/ or schools/ | 74 832 |
|  | 5 | exp Education, Special/ | 15 712 |
|  | 6 | (educat* or school* or academ* or colleg*).mp. | 1 838 718 |
|  | 7 | 4 or 5 or 6 | 1 838 718 |
|  | 8 | mainstreaming, education/ or exp education, special/ | 15 712 |
|  | 9 | (placement* or choice* or choos* or inclu* or mainstream* or special* or setting* or location* or integrat* or transition* or segregat* or type*).mp. | 11 430 083 |
|  | 10 | Choice Behavior/ | 35 582 |
|  | 11 | 8 or 9 or 10 | 11 434 510 |
|  | 12 | Students/ | 86 418 |
|  | 13 | adolescent/ or child/ | 3 234 237 |
|  | 14 | (child* or student* or adolescen* or ‘young person’ or ‘young people’ or ‘young adult*’ or youth or teen*).mp | 4 883 932 |
|  | 15 | 12 or 13 or 14 | 4 883 932 |
|  | 16 | 3 and 7 and 11 and 15 | 1141 |
| Scopus  To 2024 August week 4 | 1 | TITLE-ABS (‘Down;s syndrome’ OR ‘Down Syndrome’ OR ‘trisomy 21’ OR mongol*) | 90 985 |
|  | 2 | TITLE-ABS  (educat* OR school* OR academ* OR colleg*) | 4 439 743 |
|  | 3 | TITLE-ABS  (placement* OR choice* OR choose* OR inclu* OR mainstream* OR special* OR location* OR integrat* OR transition* OR segregat* OR type*) | 27 134 722 |
|  | 4 | TITLE-ABS (child* OR student* OR adolescen* OR ‘young person’ OR ‘young people’ OR youth* OR teen*) | 4 471 969 |
|  | 5 | 1 and 2 and 3 and 4 | 1497 |
| ERIC through EBSCOhost  1900 to 2024 August week 4 | 1 | TX(‘down syndrome’ or ‘down’s syndrome’ or ‘trisomy 21’ or mongol*) | 2910 |
|  | 2 | TX(educat* OR school* OR academ* OR colleg*) | 1 924 817 |
|  | 3 | TX(placement* OR choice* OR choose* OR inclu* OR mainstream* OR special* OR location* OR integrat* OR transition* OR segregat* OR type*) | 902 642 |
|  | 4 | TX(child* OR student* OR adolescen* OR ‘young person’ OR ‘young people’ OR youth* OR teen*) | 1 270 891 |
|  | 5 | 1 and 2 and 3 and 4 | 993 |
| ProQuest Dissertations & Theses Global  1861 to 2024 August week 4 | 1 | noft(‘down syndrome’ or ‘down’s syndrome’ or ‘trisomy 21’ or mongol*) | 5510 |
|  | 2 | noft(educat* OR school* OR academ* OR colleg*) | 1 506 222 |
|  | 3 | noft(placement* OR choice* OR choose* OR inclu* OR mainstream* OR special* OR location* OR integrat* OR transition* OR segregat* OR type*) | 2 458 105 |
|  | 4 | noft(child* OR student* OR adolescen* OR ‘young person’ OR ‘young people’ OR youth* OR teen*) | 783 702 |
|  | 5 | 1 and 2 and 3 and 4 | 699 |
|  |  | Total | 4330 |
