# Supplementary material for: Mainstream or special secondary school for the health, education, and well‐being of adolescents with Down syndrome: A systematic review
Source: Dev Med Child Neurol. 2025 Nov 4;68(6):755–66. doi: 10.1111/dmcn.70066 (PMC13160392; doi:10.1111/dmcn.70066)
Supplement: Supplementary file 4 — Table S2: Studies excluded during extended full‐text review. [file DMCN-68-755-s004.docx]

**TABLE S2:** Studies excluded during extended full-text review

| **Author (year)**  **DOI** | **Country** | **Reason for exclusion** | **Sample** | | **Study design** | **Outcome from mainstream school placement** | | | |
| --- | --- | --- | --- | --- | --- | --- | --- | --- | --- |
|  |  |  | **Mainstream** | **Special** |  | **Education** | **Social** | **Self-care** | **Health** |
| Laws et al. (2010)^1^  DOI: 10.1080/713663758 | UK | Unclear intervention: the mean age for the older group of mainstream school children was 11.6 years, which may not have allowed any attendance at secondary school. No adjustment for confounding. | 11 | 11 | Cross-sectional, difference between groups | AM (s) |  |  |  |
| Keenan (2012)^2^  DOI: 10.54014/RSEH-NVC3 | USA | Intervention group is too small (*n* = 8); no adjustment for confounding or baseline characteristics. | 8 | 12 | Cross-sectional, difference between groups | AM (s) | AM (s) |  |  |
| Foley et al. (2013)^3^  DOI: 10.1111/cch.12019 | Australia | Not enough detail to classify a comparison group; no adjustment for confounding; uncertain outcome (type of ‘post-school day occupation’). | 14 | 141 | Cross-sectional (retrospective: currently aged 15–31 years), difference between groups | AM (s) |  | AM (s) |  |
| de Graaf and de Graaf (2016)^4^  DOI: 10.1111/jppi.12161 | The Netherlands | Undefined exposure: impossible to classify whether the intervention occurred during secondary school. | 288 | 338 | Cross-sectional (retrospective intervention), difference between groups | AM (s) |  |  |  |
| Hargreaves et al. (2021)^5^  DOI: 10.1016/j.ridd.2021.104115 | UK | Uncertain outcome: participation in social activities may represent a feature of the intervention; parental satisfaction with child’s ‘progress’ is non-specific and may not refer to academic progress. | 67 | 115 | Cross-sectional, difference between groups | U | U | — | — |
| Bochner (2001)^6^  DOI: [10.1080/10349120120036314](https://doi.org/10.1080/10349120120036314) | Australia | Undefined exposure: impossible to classify whether the intervention occurred during secondary school. No control for confounding. | 12 | 18 | Cross-sectional (retrospective: currently aged 18–36 years), difference between groups | AM (0) |  |  |  |

Abbreviations: (0), no difference; AM, advantage for mainstream students; (s), statistically significant difference (*p* < 0.05); U, uncertain.

References

1. Laws G, Byrne A, Buckley S. Language and Memory Development in Children with Down Syndrome at Mainstream Schools and Special Schools: A comparison. Educational Psychology. 2000 Dec 1;20(4):447–57.

2. Keenan N. Parent-rated strengths of children and adolescents with Down syndrome. [Internet] [Doctoral Dissertation]. [Albany, New York]: University at Albany, State University of New York; 2012. Available from: https://scholarsarchive.library.albany.edu/cgi/viewcontent.cgi?article=1658&context=legacy-etd

3. Foley KR, Jacoby P, Girdler S, Bourke J, Pikora T, Lennox N, et al. Functioning and post-school transition outcomes for young people with Down syndrome. Child Care Health Dev. 2013 Nov;39(6):789–800.

4. de Graaf G, de Graaf E. Development of Self-Help, Language, and Academic Skills in Persons With Down Syndrome. Journal of Policy and Practice in Intellectual Disabilities. 2016;13(2):120–31.

5. Hargreaves S, Holton S, Baxter R, Burgoyne K. Educational experiences of pupils with Down syndrome in the UK. Research in Developmental Disabilities. 2021 Dec 1;119:104115.

6. Bochner S, Outhred L, Pieterse M. A Study of Functional Literacy Skills in Young Adults with Down Syndrome. International Journal of Disability, Development and Education. 2001 Mar 1;48(1):67–90.
